# Partners’ controlling behaviors and intimate partner sexual violence among married women in Uganda

**DOI:** 10.1186/s12889-015-1564-1

**Published:** 2015-03-04

**Authors:** Stephen Ojiambo Wandera, Betty Kwagala, Patricia Ndugga, Allen Kabagenyi

**Affiliations:** Department of Population Studies, School of Statistics and Planning, College of Business and Management Sciences, Makerere University, Kampala, Uganda; Centre for Population and Applied Statistics, Makerere University, Kampala, Uganda

**Keywords:** Intimate sexual partner violence, Empowerment, Alcohol, Controlling behaviors, Uganda

## Abstract

**Background:**

Studies on the association between partners’ controlling behaviors and intimate partner sexual violence (IPSV) in Uganda are limited. The aim of this paper was to investigate the association between IPSV and partners’ controlling behaviors among married women in Uganda.

**Methods:**

We used the 2011 Uganda Demographic and Health Survey (UDHS) data, and selected a weighted sample of 1,307 women who were in a union, out of those considered for the domestic violence module. We used chi-squared tests and multivariable logistic regressions to investigate the factors associated with IPSV, including partners’ controlling behaviors.

**Results:**

More than a quarter (27%) of women who were in a union in Uganda reported IPSV. The odds of reporting IPSV were higher among women whose partners were jealous if they talked with other men (OR = 1.81; 95% CI: 1.22-2.68), if their partners accused them of unfaithfulness (OR = 1.50; 95% CI: 1.03-2.19) and if their partners did not permit them to meet with female friends (OR = 1.63; 95% CI: 1.11-2.39). The odds of IPSV were also higher among women whose partners tried to limit contact with their family (OR = 1.73; 95% CI: 1.11-2.67) and often got drunk (OR = 1.80; 95% CI: 1.15-2.81). Finally, women who were sometimes or often afraid of their partners (OR = 1.78; 95% CI: 1.21-2.60 and OR = 1.56; 95% CI: 1.04-2.40 respectively) were more likely to report IPSV.

**Conclusion:**

In Uganda, women’s socio-economic and demographic background and empowerment had no mitigating effect on IPSV in the face of their partners’ dysfunctional behaviors. Interventions addressing IPSV should place more emphasis on reducing partners’ controlling behaviors and the prevention of problem drinking.

## Background

Sexual and gender-based violence is a major social and public health problem [1,2] with immediate and long-term negative consequences [3]. Sexual violence in particular leads to negative psychological, behavioral, physical, and reproductive health outcomes [1,4]. These include a heightened risk of HIV and sexually transmitted infections [5,6], gynecological and sexual disorders, pregnancy complications, miscarriages and low birth weight [7].

Worldwide, approximately 35% of women have experienced either physical or sexual violence, or both. Between 2006 and 2011, the percentage of Ugandan women who reported that they had ever experienced violence decreased from 39% to 28%. In the same period, the percentage of women who reported that they had experienced sexual violence in the twelve months preceding the survey decreased from 25% to 16%. The prevalence remains unacceptably high, however. In Uganda, 55% of ever married women identified intimate partners (husbands or male partners) as the perpetrators of sexual violence [8,9].

Intimate partner sexual violence (IPSV) is among the most common forms of gender-based violence in Uganda. Among women who were in a union, 27% had ever experienced IPSV from their current partner and about one in five (21%) had experienced sexual violence from an intimate partner in the 12 months prior to the survey [8].

IPSV is justified or tolerated in some cultures in South Africa [10] and Uganda [11]. This is because of associated beliefs that condone sexual violence within marriage and weak law enforcement [12,13]. In such contexts, women rarely report IPSV cases outside the family context. In Uganda, sexual violence ranked high among the types of violence where the majority of victims (65%) never sought help or told anybody [8].

In some contexts, demographic and socio-economic factors account for variations in IPSV occurrence. Significant predictors of IPSV include the woman’s age, age at first sex, type of marriage relationship, level of education, wealth status, and geographical region [2,11,14]. In rural Uganda, IPSV was associated with being young, an early age at first sex, consensual unions, and a low level of education [11]. In addition, poor wealth status significantly increases the likelihood of IPSV. The need for economic support often results in early (and possibly coerced) sex and marriage [11,12,15]. In Uganda, the likelihood of reporting IPSV was higher in the eastern and western regions than in the northern region [15].

Patriarchal notions of masculinity permeate IPSV. These notions reinforce men’s control over women and their belief in unconditional sexual entitlement in marriage. In addition, IPSV is a method of punishment for non-compliance with partners’ demands in a relationship. The occurrence of sexual violence was positively associated with women’s attitudes justifying wife beating and women earning more than their partners. Women who had decision-making autonomy had a lower likelihood of experiencing physical and sexual violence [12,16,17].

Socialization, particularly in family contexts in the formative years of life has a significant bearing on behavior during adulthood. In particular, witnessing parental violence during childhood is associated with re-victimization and an intergenerational cycle of violence for both girls and boys [12,18]. Intimate partner physical (battering) and sexual violence are closely related [19]. While several studies have established the relationship between witnessing physical intimate partner violence and experiencing physical violence [12,18,20], its association with sexual violence in Uganda is yet to be established.

Male partners’ controlling behavior in unions, their alcohol consumption and women’s fear of their partners have been associated with intimate partner violence [12,13,15,17]. Factors associated with intimate partner physical violence (IPPV) among women who are in a union in Uganda include women’s occupation; wealth status; parity; witnessing parental violence and whether women were afraid of their partners. In addition, partners’ controlling behaviors – including accusing women of unfaithfulness, denying them permission to meet their female friends, insisting on knowing their whereabouts, and getting drunk sometimes or often – were associated with IPPV [20].

Studies investigating IPPV usually combine both physical and sexual violence [6,14,21,22] yet contextual social perceptions of the two may differ [20]. On the other hand, studies that specifically address IPSV did not consider women’s empowerment and male partners’ controlling behaviors [23]. Therefore, this paper assessed the association between women’s empowerment, partners’ controlling behaviors, and intimate partner sexual violence among women who are in a union in Uganda.

## Methods

### Data source

We used the 2011 Uganda Demographic and Health Survey (UDHS) data, accessed with permission from the DHS Program website [24]. This was a cross-sectional nationally representative survey that used a stratified two-stage cluster sampling design [8] based on the sampling frame from the 2002 population and housing census [25]. A detailed description of the sampling procedure was reported in the 2011 UDHS report [8].

The sample for the domestic violence (DV) module was 2,056 ever-married women. From this sample, we extracted a weighted sample of 1,307 women who reported being in a union, for further analysis [8]. We used the domestic violence weighting variable (d005) included in the UDHS data and the Stata survey (svy) command to apply the weights during the analyses. Survey weighting is necessary to account for the complex survey design [26].

In this paper, women in a union included those who were married or cohabiting with their partners. The domestic violence module was based on a shortened and modified version of the Conflict Tactics Scale (CTS) [27]. The survey was carried out based on the World Health Organization’s (WHO) ethical and safety recommendations for research on domestic violence [28].

### Measures of outcome variable

In the DHS, intimate partner sexual violence (IPSV) was measured as having experienced any sexual violence from a current or former partner in the 12 months prior to the survey [29]. For IPSV, women in a union were asked the following questions: a) Have you ever been physically forced into unwanted sex by your husband/partner? b) Have you ever been forced into other unwanted sexual acts by your husband/partner? c) Have you ever been physically forced to perform sexual acts when you did not want to? The binary responses from these three questions were merged into an aggregate measure of intimate partner sexual violence (variable d108 in DHS), which was coded as 0 = no, and 1 = yes.

### Measures of explanatory variables

We classified independent variables into three categories: first, women’s empowerment indicators, namely participation in decision-making, attitudes justifying physical violence, and economic empowerment indicators. The second group of variables comprised husbands/partners’ controlling behavior, women’s fear of their partners, and their partners’ alcohol consumption. The third category of explanatory variables included women’s socio-demographic factors such as having witnessed parental violence themselves. This categorization was used in our earlier publication [20]. The term “partner” in this paper includes husbands as well as partners in cohabiting unions.

Five indicators in the DHS measured women’s participation in household decision-making. Earlier publications have used these indicators [20,29]. In this paper, we used all five measures of decision-making autonomy regarding who usually makes decisions about: a) how women’s earnings are used; b) women’s healthcare; c) large household purchases; d) visits to family or relatives; and e) what to do with the money the partner earns. Responses to these questions were recoded into two categories (1 = woman decides alone/jointly with partner, 0 = partner alone/others). The assumption was that women who made decisions either alone or jointly with their partners were more empowered than those in households where decisions were made by either their partners alone or other people [20,29].

Attitudes justifying physical violence were measured by questions concerning whether wife beating was justifiable if the wife: a) goes out without telling partner; b) neglects their children; c) argues with her partner and d) refuses to have sex with her partner. Responses to these variables were dichotomous (1 = “yes” or 0 = “no”). Although, some studies have aggregated these measures [29,30], we preferred individual measures of empowerment [29]; our aim was to investigate the contribution of specific empowerment measures in influencing IPSV in the Ugandan context.

As described elsewhere [20], “women’s economic empowerment included occupation and ownership of property (a house). Ownership of a house was recoded into two categories: woman alone/jointly with the partner as the empowered category and partner alone/others as the other. Ownership of a house is included because it is an important and contested asset for women in the Ugandan context” [20].

Partners’ behavior and controlling tendencies were analyzed using three variables: partners’ controlling behavior and alcohol consumption, and the women’s attitude towards their partners. To measure partners’ control, women were asked whether their present partners: a) were jealous if respondents talked with other men; b) accused them of unfaithfulness; c) did not permit them to meet female friends; d) tried to limit respondents’ contact with family and e) insisted on knowing where they were. All these variables had binary responses (0 = no and 1 = yes).

Partner’s alcohol consumption was measured by two questions: a) Does your partner drink alcohol? This was coded as binary outcome (0 = No, 1 = Yes). The second follow up question was asked to those who said yes to drinking alcohol: b) How often does (did) he get drunk: often, only sometimes, or never? The response categories were; 0 = never, 1 = often, 2 = sometimes). We generated a new measure of alcohol consumption from the second question and included those who do not drink alcohol. The new variable had four categories (0 = does not drink, 1 = drinks, but never gets drunk, 2 = drinks and sometimes gets drunk and 3 = drinks and often gets drunk). We also included women’s attitude towards their partners – whether they were afraid of their partners – in this category of variables. Women were asked if they were afraid of their partners. This was categorized as 0 = never, 1 = sometimes and 2 = most of the time.

Women’s socio-demographic characteristics included women’s age group, women’s education level, region, place of residence, wealth index, parity or number of children ever born and current marital status (married or cohabiting). Witnessing of parental intimate partner violence was measured by whether the respondent reported ever witnessing her father beating her mother (with a binary outcome of 0 = No, 1 = Yes). The analysis also included age at first sex and duration of the union.

### Statistical analyses

We used frequency distributions to describe the characteristics of the women. Cross-tabulations with Pearson's chi-squared (*χ*^2^) tests were used to examine the associations between IPSV and women’s empowerment (economic empowerment, attitudes justifying physical violence, and decision-making autonomy), partners’ behaviors, and women’s socio-demographic factors. The level of statistical significance using p-values was set at *p* <0.05.

Finally, we conducted multivariable logistic regression analyses to examine the association between IPSV and the selected explanatory variables whose p-values were less than 0.05 during the chi-square tests. We presented the results in the form of Odds Ratios (OR) and their 95% confidence intervals. The level of statistical significance using p-values was set at *p* < 0.05. We estimated three models during the multivariable analysis. In the first step, IPSV was modeled with decision-making indicators and attitudes justifying wife beating. In the second model, we added partners’ controlling behaviors and women’s fear of their partners. In the final model, we adjusted for socio-demographic factors. We weighted all of the analyses to take into consideration the survey design, clustering and stratification [26].

## Results

### Distribution of respondents by socio-demographic characteristics and measures of empowerment

From Table 1, 41% of the women were aged 25–34 years. There was nearly an even distribution of women by region, although more than a quarter (28%) were from the central region. Most (84%) of the women were rural residents. Most (70%) engaged in sex before they were 18 years. More than half (55%) were married and had been in a union for over 10 years (55%). Half (50%) of the women reported giving birth to between 1 and 4 children in their lifetime. More than half (60%) had primary education and 41% were from the richer and richest wealth quintiles. Close to half (46%) had witnessed their fathers beat their mothers during childhood.

**Table 1:** **Percentage distribution of married women socio-demographics and experience of intimate partner sexual violence (IPSV) in Uganda (DHS 2011)**

**Variables**	**% of women**	**Frequency**	**% reported IPSV**	**p-value**
**Age group**				0.459
15-24	29.7	388	24.6	
25-34	40.6	531	28.7	
35+	29.7	388	25.4	
**Region**				**0.006**
Central	28.0	366	24.9	
Eastern	26.3	344	34.4	
Northern	19.2	251	26.0	
Western	26.4	346	20.7	
**Residence**				0.262
Urban	16.4	214	23.0	
Rural	83.6	1093	27.2	
**Age at first sex**				**0.038**
18+	29.9	391	20.8	
<14	22.8	298	29.2	
15-17	47.3	618	28.8	
**Current marital status**				0.119
Married	55.3	723	24.4	
Cohabiting	44.7	584	29.1	
**Duration of union in years**				0.537
0-4	24.1	315	22.3	
5-9	20.8	272	27.5	
10-14	18.7	244	27.6	
15-19	15.3	199	26.3	
20+	21.2	277	29.5	
**Parity**				0.297
0	6.7	87	19.7	
1-4	50.0	653	25.3	
5+	43.4	567	29.0	
**Women’s education level**				**0.017**
No education	17.0	222	24.1	
Primary	60.1	785	29.9	
Secondary	22.9	299	19.5	
**Wealth index**				0.105
Poorest	18.6	243	30.3	
Poorer	19.9	260	29.3	
Middle	20.1	262	28.1	
Richer	19.5	255	26.9	
Richest	21.9	287	19.0	
**Woman’s father ever beat her mother**				**0.004**
No	43.0	562	22.1	
Yes	45.9	600	31.7	
Don’t know	11.1	144	22.2	
**Women’s occupation**				0.056
Not working	23.7	310	24.3	
Professional/technical/managerial	4.0	52	9.1	
Agriculture	53.4	698	28.5	
Sales	18.9	247	27.5	
**Woman owns house alone or jointly**				0.402
No	42.7	558	25.1	
Yes	57.3	749	27.6	
**Woman decides alone or jointly on:**				
**Spending her income**				0.406
No	56.2	734	25.4	
Yes	43.8	573	27.9	
**Her own healthcare**				0.506
No	40.8	533	27.7	
Yes	59.2	774	25.7	
**Large household purchases**				0.402
No	43.8	572	27.9	
Yes	56.2	735	25.4	
**Visits to family**				0.177
No	42.4	555	28.9	
Yes	57.6	752	24.8	
**What to do with partner’s income**				**0.003**
No	54.3	710	30.6	
Yes	45.7	597	21.7	
**Beating justified if wife:**				
**Goes out without telling husband**				0.457
No	59.1	772	25.6	
Yes	40.9	535	27.8	
**Neglects the children**				**0.021**
No	52.3	683	23.3	
Yes	47.7	624	30.1	
**Argues with husband**				**0.014**
No	69.8	912	24.1	
Yes	30.2	395	32.1	
**Refuses to have sex with husband**				0.863
No	76.2	996	26.4	
Yes	23.8	311	27.0	
**Burns the food**				**0.046**
No	85.0	1112	25.4	
Yes	15.0	195	33.1	
**Total**	100.0	1307	26.5	

Concerning measures of women’s empowerment, just over half (53%) of the women were engaged in agriculture, more than half (57%) reported ownership of a house either alone or jointly. Participation in household decision-making among women in a union was as follows: how women’s earnings were spent (44%); women’s own healthcare (59%); large household purchases (56%); visits to women’s family (58%); and how their partners’ earnings were used (46%).

The prevalence of attitudes justifying physical violence varied. Wife beating was justified if the woman: went out without telling her partner (41%); neglected their children (48%); argued with her partner (30%); refused sex with her partner (24%); or burnt food (15%). Just over one in four (27%) women in a union reported having experienced IPSV in the 12 months preceding the survey.

### The association between IPSV and women’s socio-demographic characteristics and empowerment indicators

Results of the cross tabulations in Table 1 show that region, age at first sex, women’s level of education, and whether the woman’s father beat her mother were significantly associated with IPSV. Among the measures of empowerment, IPSV was only significantly associated with decision-making regarding the partner’s income, and with attitudes towards wife beating only if the woman neglected their children, argued with her partner, or burnt food. However, some of the key measures of economic empowerment, such as property ownership and decision-making concerning large purchases were not significantly associated with IPSV (see Table 1).

### Distribution of respondents by their’ partners’ background and behavioral factors

Table 2 presents male partners’ background characteristics, their behavior (controlling tendencies and alcohol consumption) and women’s fear of their partners. Over half (55%) of the partners were aged 35 years and above. Over a third (36%) had secondary or higher education. Concerning partners’ controlling behavior, over half of the women reported that their partners were jealous if they talked with men (56%), and insisted on knowing where they were (55%). Less than one third reported that their partners accused them of unfaithfulness, did not permit them to meet female friends, and limited their contact with family (32%, 26% and 18% respectively). Four in ten (40%) of the women had partners who got drunk and 46% of the women were afraid of their partners.

**Table 2:** **Percentage distribution of married women by male partners’ related factors, controlling behaviors and experience of IPSV in Uganda (DHS 2011)**

**Variables**	**% of women**	**Frequency**	**% reported IPSV**	**p-value**
**Partners’ age group**				0.405
15-24	9.9	130	22.2	
25-34	35	458	25.3	
35-44	31.8	416	30.0	
45+	23.2	303	25.4	
**Partner’s education level**				0.077
No education	10.1	132	25.6	
Primary	54	706	29.9	
Secondary	27.5	360	22.6	
Higher	8.4	109	18.8	
**Husband/partner:**				
**Jealous if woman talks with men**				**0.000**
No	43.6	570	15.3	
Yes	56.4	737	35.2	
**Accuses her of unfaithfulness**				**0.000**
No	68.1	890	19.9	
Yes	31.9	418	40.6	
**Does not permit her to meet female friends**				**0.000**
No	73.8	965	21.1	
Yes	26.2	342	41.8	
**Limits her contact with family**				**0.000**
No	81.5	1066	21.9	
Yes	18.5	241	46.8	
**Insists on knowing where she is**				**0.000**
No	45.2	591	18.4	
Yes	54.8	716	33.2	
**Partner drinks alcohol**				**0.000**
No	55.0	719	25.4	
Yes, but never gets drunk	4.8	63	8.3	
Yes, sometimes gets drunk	25.0	326	21.8	
Yes, often gets drunk	15.2	199	44.1	
**Woman afraid of partner**				**0.000**
Never	54.4	711	18.2	
Sometimes	27.1	354	35.1	
Often	18.5	242	38.5	
**Total**	100	1307	26.5	

### The association between IPSV among women in a union and partners’ behaviors

In Table 2, all measures of male partners’ controlling behaviors, alcohol consumption, and women’s attitudes towards their partners were strongly associated with reporting IPSV. IPSV was higher among women whose partners were jealous when they talked to other men (35%), accused them of unfaithfulness (41%), denied them permission to meet female friends (42%), limited contact with family (46%), and insisted on knowing their whereabouts (33%). IPSV was higher among women whose partners often got drunk (44%) and among women who were often afraid of their partners (39%). Partner’s age and level of education were not significantly associated with IPSV.

### Multivariable results

Table 3 shows the results of logistic regression of IPSV controlling for empowerment indicators and selected explanatory variables. In the first model, we adjusted for decision-making and attitudes justifying wife beating. Woman’s participation in decision-making concerning the partner’s income was the only significant predictor of IPSV. Women who participated in decision-making concerning their partners’ incomes had reduced odds (OR = 0.63; 95% CI: 0.47-0.86) of experiencing IPSV in comparison to those who did not.

**Table 3:** **Results of logistic regression of IPSV and empowerment indicators controlling for women’s socio-demographic factors and male partners’ controlling behaviors in Uganda (DHS 2011)**

**Experienced intimate partner sexual violence (IPSV) in the last 12 months preceding the survey**						
	**Model (1)**		**Model (2)**		**Model (3)**	
**Variables**	**ORs**	**95% CI**	**ORs**	**95% CI**	**ORs**	**95% CI**
**Woman decides on partner’s income alone or jointly (RC = No)**	0.633^**^	[0.47-0.86]	0.743	[0.53-1.04]	0.774	[0.55-1.08]
**Beating justified if wife:**						
Neglects the children (RC = No)	1.224	[0.87-1.71]	1.259	[0.88-1.79]	1.260	[0.88-1.80]
Argues with husband (RC = No)	1.247	[0.86-1.80]	1.016	[0.69-1.50]	0.945	[0.63-1.41]
Burns the food (RC = No)	1.195	[0.79-1.81]	1.094	[0.70-1.71]	1.051	[0.67-1.65]
**Husband/partner:**						
Jealous if woman talks with other men (RC = No)			1.930^**^	[1.28-2.90]	1.808^**^	[1.22-2.68]
Accuses respondent of unfaithfulness (RC = No)			1.455	[1.00-2.12]	1.501^*^	[1.03-2.19]
Does not permit respondent to meet female friends (RC = No)			1.530^*^	[1.03-2.27]	1.630^*^	[1.11-2.39]
Tries to limit respondent’s contact with family (RC = No)			1.815^**^	[1.16-2.83]	1.725^*^	[1.11-2.67]
Insists on knowing where respondent is (RC = No)			0.968	[0.66-1.42]	0.940	[0.64-1.37]
**Partner drinks alcohol** (RC = No)						
Yes, but never gets drunk			0.294^**^	[0.13-0.66]	0.313^**^	[0.14-0.71]
Yes, sometimes gets drunk			0.688	[0.46-1.04]	0.717	[0.47-1.10]
Yes, often gets drunk			1.803^**^	[1.18-2.77]	1.800^**^	[1.15-2.81]
**Woman afraid of partner** (RC = Never)						
Sometimes			1.819^**^	[1.25-2.65]	1.777^**^	[1.21-2.60]
Often			1.593^*^	[1.04-2.44]	1.578^*^	[1.04-2.40]
**Region** (RC = Central)						
Eastern					1.051	[0.65-1.69]
Northern					0.985	[0.62-1.57]
Western					0.754	[0.46-1.24]
**Women’s education level** (RC = None)						
Primary					1.269	[0.82-1.96]
Secondary					0.882	[0.46-1.68]
**Woman’s father ever beat her mother** (RC = No)						
Yes					1.347	[0.96-1.88]
Don’t know					0.964	[0.55-1.69]
**Age at first sex** (RC = 18+ years)						
<14 years					0.949	[0.59-1.54]
15-17 years					1.159	[0.78-1.73]
**Weighted sample**	**1307**		**1307**		**1307**	

ORs = Odds Ratios; CI = Confidence Interval; RC = Reference Category; *p < 0.05- **p < 0.01- ***p < 0.001.

In the second model, we added partners’ behaviors and women’s fear of their partners. Partners’ behaviors and women’s fear of their partner weakened women’s participation in decision-making concerning their partners’ income. Among partner’s behaviors, significant predictors of IPSV were if the partner was jealous if the woman talked with other men, did not permit the respondent to meet female friends, tried to limit the woman’s contact with family, and the frequency of the partner’s excessive alcohol consumption. Whether the woman was afraid of her partner was also significantly associated with IPSV. The odds of reporting IPSV were higher among women whose partners were jealous if they talked to other men (OR = 1.93; 95% CI: 1.28-2.90), did not permit the respondent to meet female friends (OR = 1.53; 95% CI: 1.03-2.27), and tried to limit her contact with family (OR = 1.82; 95% CI: 1.16-2.83). Women whose partners often got drunk had increased odds (OR = 1.80; 95% CI: 1.18-2.77) of reporting IPSV. Likewise, woman who were sometimes or often afraid of their partners were more likely to report IPSV (OR = 1.82; 95% CI: 1.25-2.65 for women who were sometimes afraid, and OR = 1.59; 95% CI: 1.04-2.44 for those who were often afraid).

In the final model, we adjusted for socio-demographic factors. While most of the partners’ behaviors and women’s attitude towards their partners retained their significance, women’s empowerment and socio-demographic variables were not significant. The odds of reporting IPSV were higher among women whose partners were jealous if they talked with other men (OR = 1.81; 95% CI: 1.22-2.68), accused them of unfaithfulness (OR = 1.50; 95% CI: 1.03-2.19) and did not permit them to meet with their female friends (OR = 1.63; 95% CI: 1.11-2.39). The odds of IPSV were also higher among women whose partners tried to limit their contact with family (OR = 1.73; 95% CI: 1.11-2.67) and often got drunk (OR = 1.80; 95% CI: 1.15-2.81). Women who were sometimes or often afraid of their partners had increased odds of reporting IPSV (OR = 1.78; 95% CI: 1.21-2.60 and OR = 1.58; 95% CI: 1.04-2.40 respectively).

## Discussion

The aim of this study was to examine the association between women’s empowerment, partners’ controlling behaviors and IPSV. The prevalence of IPSV among women in a union was high.

Male partners’ controlling behaviors were the main predictors of IPSV among women in a union in Uganda. Being jealous if their partner talked with other men, accusing her of unfaithfulness, not permitting her to meet with female friends, and trying to limit her contact with family, were associated with IPSV. Other studies elsewhere have reported an association between male partners’ controlling behaviors and IPSV [12,13]. In the case of IPSV, women’s empowerment as measured by the UDHS [8,9] and their socio-demographic characteristics were not significant predictors of IPSV after adjusting for partners’ controlling behaviors. It is apparent that IPSV would occur irrespective of women’s empowerment or socio-demographic background, if their partners exhibit excessive controlling tendencies and alcohol consumption.

Women’s fear of their partners (which is most likely a result of partners’ abusive behaviors) was associated with IPSV. Women who were afraid of their partners were less likely to say no to the sexual advances of their partners due to the threat of physical violence [20]. As noted earlier, their fear could also be a result of IPSV [19]. Excessive alcohol consumption (often getting drunk) remains a significant predictor of both intimate partner sexual and physical violence [20]. This finding has been reported in South Africa [12] and Rwanda [17].

In this paper, some of the predictors of IPSV varied from those of IPPV in other studies [12,13,18,20]. Significant predictors of IPPV, namely parity, wealth status, woman’s occupation, experience of parental violence, and the partner insisting on knowing his wife’s whereabouts did not have a significant relationship with IPSV. On the other hand, the significant predictors of IPSV in this paper - the partner being jealous if the woman talked with other men, and the partner trying to limit the woman’s contact with her family - did not predict the occurrence of IPPV in another Ugandan study [20]. Our findings confirm that there are variations between predictors of IPSV and IPPV. The common predictors of IPPV and IPSV were accusing wives of unfaithfulness, not permitting women to meet female friends, getting drunk, and women’s fear of their partners [20].

While witnessing parental physical violence was a significant predictor of IPPV [18,20], it did not predict the occurrence of IPSV. Although it is possible that witnessing parental IPSV could result in IPSV, the UDHS did not address this question (perhaps owing to its sensitivity).

None of the socio-demographic factors considered (age of the woman, age at first sex, type of union, level of education and region), were significant predictors of IPSV among women in a union Uganda. This is contrary to findings elsewhere in Africa [11,13,17]. While the duration of the union and women’s control over their incomes were significant predictors of IPSV in Rwanda [17], this was not the case in Uganda. In addition, neither women’s empowerment (including their participation in household decision-making, their attitudes towards wife beating, their control over their incomes, and their occupation) nor their household wealth status, were significant predictors of IPSV in the context of their partners’ dysfunctional behaviors.

## Conclusions

In Uganda, women’s socio-economic and demographic backgrounds and empowerment had no mitigating effect on IPSV in the face of their partners’ dysfunctional behaviours. Interventions addressing IPSV should target men, placing more emphasis on addressing the root causes of their controlling behavioral tendencies, and the prevention of problem drinking.
